# The Impact of COVID-19 Safety Recommendations on Adults Age 60 and Older: A Qualitative Study

**DOI:** 10.1093/geroni/igaa057.3506

**Published:** 2020-12-16

**Authors:** Kerstin Emerson, Deborah Kim, George Mois, Jenay Beer

**Affiliations:** 1 University of Georgia, Athens, Georgia, United States; 2 University of Georgia, Lawrenceville, Georgia, United States; 3 Institute of Gerontology, Athens, Georgia, United States

## Abstract

In March 2020, the United States Centers for Disease Control (CDC) began recommending social distancing and sheltering in place, in particular for older adults. This resulted in many older adults staying at home for long periods of time in relative isolation. Because there is little prior evidence of the emotional impact that this has on older adults, we conducted an exploratory qualitative study on how older adults felt during the first three to five weeks of the CDC recommendations. We fielded a web-based cross-sectional survey. Our analytic sample consisted of 673 respondents aged 60 and older who respondent to the prompt: “How are you feeling during this time of social distancing?”. We used a thematic bottom-up qualitative analysis, via MAXQDA, to analyze segments into general affect codes and detailed emotion subcodes, as well as coping mechanisms. Results showed that while many older adults reported neutral (9%) or positive (9%) affect, a larger proportion reported negative affect (42%) or reported mixed affect (35%). The most common negative emotions mentioned were anxiety and loneliness/boredom, while the most common positive emotions mentioned were optimism and feeling grateful. The most common coping mechanisms reported by participants included making life adjustments, keeping busy, prayer/spirituality, and mediation/mindfulness. This study provides an initial understanding into how older adults experienced and coped with the first stages of restricting social interactions. If social distancing continues to be a recommended disease-containment strategy, information about how older adults are coping can be critical for public health interventions. Implications will be discussed.

